# The impact of rumination on fibromyalgia pain after physical activity: an experimental study

**DOI:** 10.1038/s41598-023-47414-z

**Published:** 2023-11-22

**Authors:** Jérémy Fonseca das Neves, Monika Kornacka, Eric Serra, Noémie Rollin, Thierry Kosinski, Virginie Maréchal, Louis Jehel, Stéphane Rusinek

**Affiliations:** 1 Psychiatrie de Liaison, Centre Hospitalier Universitaire Amiens-Picardie, Amiens, France; 2 Emotion Cognition Lab, SWPS University, Technikow 9, 40-326 Katowice, Poland; 3 Centre d’étude et de Traitement de la Douleur, Centre Hospitalier Universitaire Amiens-Picardie, Amiens, France; 4 Univ. Lille, ULR 4072 - PSITEC - Psychologie: Interactions Temps Émotions Cognition, F-59000 Lille, France; 5 Present Address: UFR Santé, Université de Picardie Jules Verne, Amiens, France; 6 Consultation de la Douleur, Centre Hospitalier de Soissons, Soissons, France; 7 Equipe MOODS-IPSOM, U1018, CESP/INSERM, 94807 Villejuif Cedex, France

**Keywords:** Health care, Risk factors, Psychology

## Abstract

Some fibromyalgia (FM) patients engage in rumination (i.e. a chain of repetitive, passive and relatively uncontrollable thoughts focused on negative content) to cope with the pain and discomfort of daily activities. The partial model of rumination in chronic pain suggests that rumination processes may play a causal role in maintaining pain. Rumination might also be one of the key factors interfering with the reestablishment of adapted physical activity. The objective of this study was to test how rumination vs. distraction induction influence FM patients’ pain intensity, discomfort linked to pain, and affect after physical activity. Forty-seven participants with a diagnosis of FM were randomly assigned to undergo distraction induction vs. rumination induction after performing a physical activity in ecological setting. Their pain intensity, pain-related discomfort, and affect were measured at the baseline, after physical activity, and after rumination versus distraction induction. A series of mixed-design ANOVAs showed that rumination induction after physical activity impairs patients’ recovery in terms of pain intensity and discomfort, but not affect, as compared to the distraction condition. In conclusion, participants with fibromyalgia who engage in rumination following a physical activity recover less from their pain experience as compared to distraction induction. These results are consistent with the partial model of rumination in chronic pain and support the idea that rumination may play a causal role in the development and maintenance of pain.

## Introduction

Fibromyalgia (FM) is a chronic diffuse dysfunctional pain syndrome that affects around 2% of the population of Western countries^1–4^. Systematic literature reviews and epidemiologic studies indicate that fibromyalgia is associated with a deterioration in quality of life^5^, increased mortality^6^ and with a strong psychopathological comorbidity, particularly with depression^7,8^. Although its pathogenic mechanisms are not fully explored, the literature suggests that one of the main mechanisms of FM is linked to a deregulation of pain control at different levels of the nervous system^9^. According to the evidence-based recommendations, optimal treatment of FM requires a transdisciplinary approach combining pharmacological and non-pharmacological treatments including resuming of physical activities^10^.

However, resuming adapted physical activities is challenging for FM patients from a clinical point of view^11,12^. On the one hand, patients are often aware that adapted physical activity is crucial to the therapeutic process, but on the other hand their previous experiences of physical activity causing discomfort and pain sustain a vicious circle of fear and avoidance^13,14^. According to the fear avoidance model (FAM^15^), pain can lead to a catastrophizing thinking about movement and activities, which can develop into an excessive, irrational and debilitating fear of movement (kinesiophobia) and having the feeling that one is more prone to experience a painful injury or re-injury^15^. This feeling might lead to avoidance of both, movement in general and physical activity, and avoidance in turn, leads to more fear^15^. In spite of the lack of consensus on the theoretical background of pain catastrophizing (i.e. a form of abstract and intrusive repetitive negative thinking, which is difficult to disengage from^16,17^), rumination about the consequences of physical activity, negative affect and pain seems to be one of the key mechanisms involved in this process (the partial model of rumination in pain^18^). Thus, addressing rumination while resuming physical activity in FM patients might be a promising path for improving adapted physical activity compliance and consequently enhancing FM patients’ wellbeing. Although the link between pain and rumination was already shown in several qualitative (e.g.,^19^), correlational (for a review see:^20^) or experimental (e.g.,^21^) studies, the impact of rumination on pain was not experimentally tested in the context of physical activity.

Rumination, defined as a chain of repetitive, passive and relatively uncontrollable thoughts focused on negative content (including pain and its consequences in the FM context)^22^, might have a causal role in decreasing physical activity in chronic pain for several reasons. On the one hand, rumination has been shown to be associated with higher pain intensity^18,23,24^ and poorer therapeutic outcomes^25^. A neuroimaging study suggested that rumination might be linked to impaired anticipatory treatment of aversive stimuli related to pain in healthy population resulting in exaggerated response of pain^21^. This mechanisms might also potentially explain how rumination affects avoidant behaviors by modifying the expected outcomes of pain. On the other hand, qualitative research^19^ along with the conclusions from a literature review^26^ suggest that rumination might be also triggered by the experience of pain itself. Moreover, it seems that patients with chronic pain may have positive metacognitive beliefs about rumination—they tend to think that rumination might help to cope and serve as a problem solving strategy^18,19^. This kind of metacognitive beliefs might also mediate the positive relation between pain intensity and catastrophizing^27^.

Additionally, rumination theory^28^ sheds some light on the link between rumination and avoidance that can be applied to the perspective of chronic pain and kinesiophobia. Watkins and Nolen-Hoeksema^28^ suggest that maladaptive, abstract rumination might be considered as emotional avoidance by disconnecting the patient from their current experience; this mechanism might provide momentary relief, but will have deleterious long-term consequences (e.g. increasing depression and/or anxiety) as it impairs emotion regulation and might be also linked to deregulation in the level of goal and action identification, i.e. flexibly adjusting the level of abstract vs. concrete processing to the ongoing situation^28,29^. The overuse of abstract processing, on its turn, might result in decreased general motivation and commitment for pursuing a goal^30^, which might be of a particularly relevance in the context of resuming adapted physical activity in chronic pain patients. Moreover, experiential avoidance (i.e. deliberate effort to alter the form or frequency of aversive experience such as negative emotions or unwanted cognitions) has been experimentally shown to reduce the pain tolerance^31^. This mechanism, in line with patients’ positive metacognitive beliefs, might explain how rumination can become a preferential strategy in pain regulation and reduce the use of other emotion regulation strategies^18^ such as distraction which could be considered as a more effective strategy to cope with pain^32^ and as an adaptive alternative for rumination^30^. Finally, the literature provides some evidence that rumination might be a mediator or moderator between certain risk factors of pain (e.g., gender^33^; pain anticipation^21^; maladaptive coping strategies^34^; functional disabilities^35^) and pain itself. Furthermore, rumination might play a mediator role in the link between pain and psychological distress^36^ and be a mediator between pain and depressive symptoms in a non-clinical population suffering from pain^37^. It can also mediate the negative relation between protective factors (e.g. mindfulness) and depression in chronic pain patients^38^. Thus, addressing rumination in FM patients might also potentially reduce the risk of other comorbid psychological disorders.

In sum, the use of rumination to respond to the discomfort of daily physical activities might maintain a higher pain after exercise compared to other strategies (e.g., distraction) through cognitive and attentional processes, but also impair long term emotion regulation through the mechanism of experiential avoidance. However, the direct impact of rumination as a regulation strategy in FM has never previously been tested in experimental settings. Exploring this path seems to be particularly appealing as, according to a recent study, FM patients might be more prone to ruminative style of thinking^39^ and to anger rumination^40^ compared to non FM chronic pain patients.

Thus, the aim of this study was to test how rumination induction versus distraction induction impact the evolution of pain (pain intensity and discomfort caused by pain) and affect in FM patients after they performed an ecological physical activity (climbing stairs). To our knowledge, this is the first time that this kind of experimental protocol has been used in the context of the evaluation of repetitive negative thinking in FM. We hypothesized that patients using a rumination strategy just after an uncomfortable physical activity will show a poorer evolution of pain, subjective discomfort and negative affect as compared to patients using a distraction strategy.

## Materials and method

### Study design

**Figure 1:**
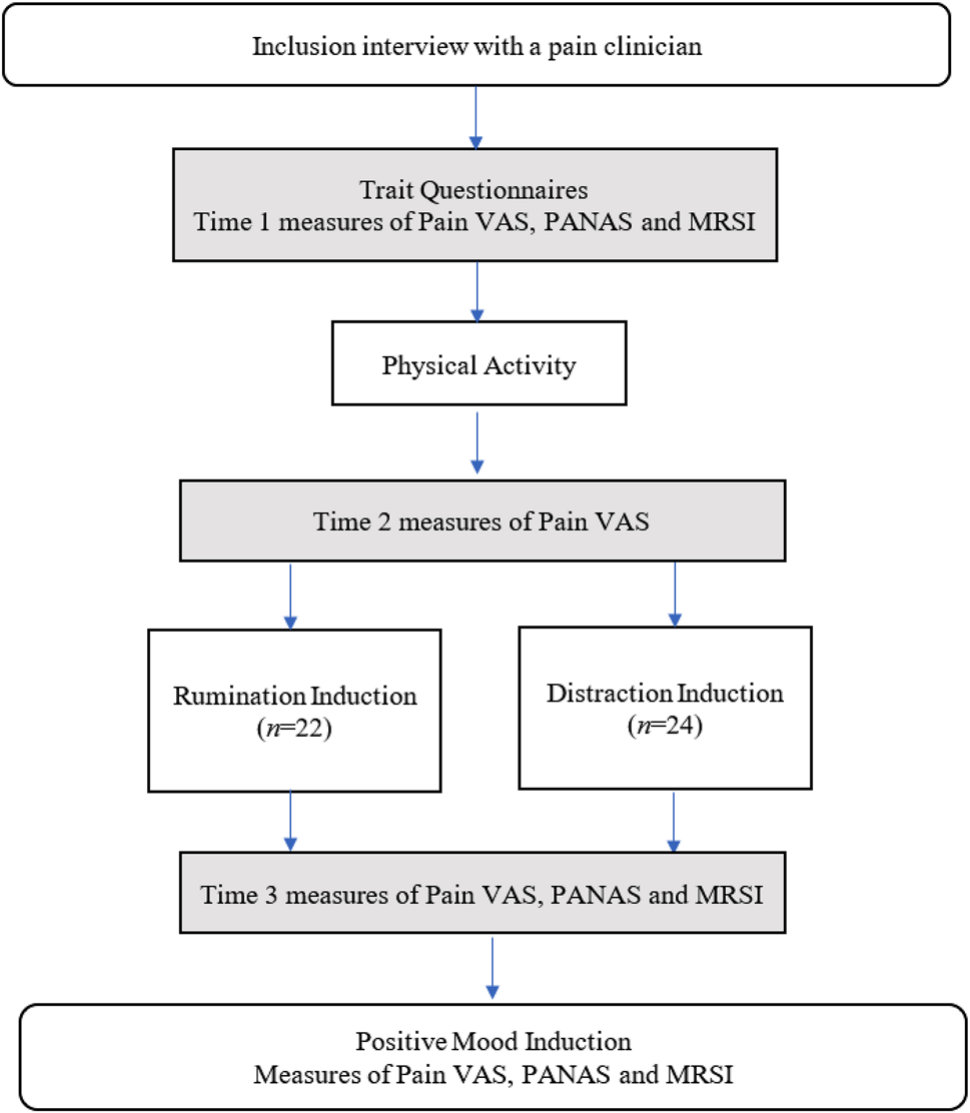
Schematic procedure flow. *Note*: Pain Vas – Pain Analogue Visual Scale ; PANAS – Positive and Negative Affect Schedule, MRSI – Momentary Rumination State Inventory.

Participants were divided randomly into two experimental groups (rumination vs. distraction induction). The dependent variables (affect and state rumination) were measured at the baseline and after experimental induction (time 1 and 3 in Fig. 1). The pain (intensity and discomfort) was measured at the baseline, after physical activity, and after rumination vs. distraction induction (see Fig. 1). Thus, we expected interaction between measure time (1 and 3 for affect and state rumination; 2 and 3 for pain) and group in the mixed-designed 2 × 2 ANOVA analysis. The total sample size required to test mixed-design 2 × 2 ANOVA, computed prior to the experiment through GPower 3.1.9.4.^41^ to detect a medium effect size, with a power of 0.90, was 46 participants.

This research was approved by Comités de protection des personnes (French central research ethics committees), CPP Centre-Ouest I nr. 2018T2-16 dated 08/28/2018. All methods described below were performed in accordance with the relevant guidelines and the study was run in accordance with the Declaration of Helsinki.

### Participants and procedure

Forty-seven adult patients (44 female, 3 male; mean age = 50.6, *SD* = 8.1) meeting the 1990 American College of Rheumatology (ACR) criteria^1^ for FM were recruited by a pain physician and psychiatrist in Amiens University Hospital Center (France) during a usual consultation.

The inclusion criteria were as follows: meeting the FM ACR 1990 criteria^1^, with stable treatment for at least 1 month, ability to understand and read French. The exclusion criteria were: being deprived of liberty or being a protected adult (under guardianship or curatorship), suffering from psychosis or severe depression or severe anxiety, or being characterized by impulsivity disenabling given participant to complete the questionnaires or take part in the study, according to the clinician's assessment.

Participants were randomly assigned to two experimental groups: a rumination induction group (*n* = 22; 20 women; mean time from the FM diagnosis in years = 5.18, *SD* = 5.05; mean education level in years after obtaining A level = 1.77, *SD* = 0.81) and distraction induction group (*n* = 25; 24 women; mean time from the FM diagnosis in years = 6.64, *SD* = 5.20; mean education level = 1.76, *SD* = 0.72). The two groups did not differ in age (*t*_*(45)*_ = -0.652, *p* = 0.518), time from the diagnosis of FM in years (*t*_*(45)*_ = -0.972, *p* = 0.336) or in education level (*χ*^*2*^_*(2, N*=*47)*_ = 0.81, *p* = 0.451). There was no significant difference between the two groups in terms of tendency for rumination (*t*_*(45)*_ = 0.25, *p* = 0.803), in terms of anxiety (*t*_*(45)*_ = 0.57, *p* = 0.573), in level of depression (*t*_*(45)*_ = 0.06, *p* = 0.961), or disability related to FM (*t*_*(45)*_ = − 1.5. *p* = 0.132). Descriptive statistics for each group are presented in the Table 1.

**Table 1:** Descriptive statistics of questionnaire measures and age for each experimental group.

Variable	Rumination induction group	Distraction induction group
	Mean (SD)	Mean (SD)
Age	49.77 (8.32)	51.32 (7.95)
Rumination (PTQ)	23.95 (10.02)	23.20 (10.49)
Anxiety (HADS)	13.00 (3.95)	12.32 (4.22)
Depression (HADS)	9.95 (3.93)	9.88 (4.26)
Disability related to FM (FIQ)	49.23 (12.78)	54.25 (9.61)

*PTQ* Perseverative Thinking Questionnaire, *HADS* Hospital Anxiety and Depression Scale, *FIQ* Fibromyalgia Impact Questionnaire, *FM* Fibromyalgia.

Patients were recruited during a standard consultation with their pain physician (being also a psychiatrist) at the pain consultation unit of the Amiens Hospital Center, France. The whole recruitment was performed by one practitioner. Each patient fulfilling the inclusion criteria was systematically invited to participate in the study by the investigator. The study was run by two investigators—clinical psychologists. Before any examination related to the research, the investigator provided all the necessary information about the study and answered potential questions concerning the objective, nature, and constraints of the research. He/she also clarified patient rights and verified eligibility criteria. Before starting the experiment, the informed consent was obtained from all participants and a copy of the information note and consent form was given to each participant.

After inclusion, participants were randomly assigned to one or other study group (Rumination group; Distraction group). This randomization was conducted by one of the collaborating members according to a randomization list generated randomly with an algorithm created using MS Excel software. The investigator had no access to this randomization at any time.

First, the participants filled in trait questionnaires in order to control for baseline group differences in trait tendency to ruminate (PTQ^42,43^), disability related to FM (FIQ^44,45^) anxiety and depression (HADS^46,47^). After filling in the questionnaires, participants filled in a baseline measure of Pain on visual analogue scale (VAS) and affect and they underwent pain activation by performing physical activity. After reevaluating pain, they underwent rumination vs. distraction induction followed by the same state measures as in Time 1. It is important to note that in Time 2 only measure of Pain was performed in order to reduce the number of measures and shorten the gap between pain activation and rumination induction. The procedure flow is presented in Fig. 1.

After the final measures, participants underwent a positive mood induction procedure (by presentation of positive images from the International Affective Picture System^48^). This induction was not a part of the experiment and was performed for ethical reasons in order to restore positive affect. A new evaluation of pain and affect was performed in order to check whether those variables’ level returned to baseline (this measure was not included in the analysis).

It is important to underline that the distraction vs. rumination induction procedure did not allow for double-blind work. The differences between distraction and rumination were possible to identify by the investigator performing the experiment from the start of rumination vs. distraction induction. To ensure a form of blinding for the patients, they were informed that we were testing pain regulation strategies without the strategy they were using being explicitly named and they were not aware of the strategy of the other group. In addition, the instructions were given in such a way as not to imply that one strategy was more effective than the other.

## Materials

### Trait questionnaires

#### Perseverative thinking questionnaire^42,43^

The trait tendency to use rumination was assessed through a 15-item Perseverative Thinking Questionnaire. The scale measures ruminative thoughts, regardless of their content, from a transdiagnostic perspective (e.g., “My thoughts repeat themselves”). Participants responded on a five-point Likert scale, from “0” (never) to “4” (almost always). The total score range between 0 and 50. A higher score on PTQ reflects a higher level of rumination. The internal consistency of the scale in the present study was excellent (*α* = 0.95).

#### Fibromyalgia impact questionnaire^44,45^

To assess disability related to FM, the Fibromyalgia Impact Questionnaire was used. It is a valid instrument for measuring the impairement associated with FM in the daily lives of patients. Items in the part 1 of the questionnaire (item 1a to 1j) exploring functional abilities ranged from 0 to 3 (the average of the questions to which the patient answered). Patients responded on a Likert scale from 0 (always) to 3 (never) assessing their ability to perform given activity during the previous week. (e.g., “during the last week, could you do the market?). In part 2 (items 2 and 3) patients reported the number of days they felt well and the number of days they missed work during the previous week. In the part 3 (items 4 to 10) patients are asked to report their current condition (e.g.“over the past seven days how much pain did you have?”) on the visual analogue scales of 10 cm. The total score ranges from 0 to 100. A higher score on the Fibromyalgia Impact Questionnaire reflects a higher level of functional disabilities related to FM. Internal consistency of the questionnaire in the present study was good (*α* = 0.84).

#### Hospital anxiety and depression scale^46,47^

The HADS enabled screening for anxiety and depressive disorders. It comprised 14 items rated from 0 (never) to 3 (almost always). HADS contained seven questions assessing anxiety (e.g., “I feel tense or upset”) and seven assessing depression (e.g.,“I’m in a good mood”). A higher score on each dimension reflected a higher level of anxiety or depression respectively. For each dimension the total score range between 0 and 21. Anxiety or Depression is considered clinically significant from the cut-off score of 11. Internal consistency was satisfactory for Anxiety (*α* = 0.77) and for Depression dimensions (*α* = 0.80).

### Experimental measures and materials

#### Physical activity induction in ecological setting

Participants were asked to climb stairs at sustained pace, in the presence of a member of the research team/clinician who regularly assessed patient’s pain intensity. The objective was to obtain an increase in pain of at least 10% on the VAS by engaging patients in moderate-intensity physical activity in an ecological setting as similar as possible to those patients experience in their daily lives. In agreement with the medical team of the hospital pain consultation unit, we estimated that a 10% increase in pain following this activity would be a good indicator of the significance of this activity. An additional limit of 6 floors was added after a pre-test performed on 10 participants. We had initially judged that going up one floor at a sustained pace would be enough to achieve this objective. However, we could see that, for the first 10 participants who served as a pretest, even if they considered the exercise physically significant, we only obtained the desired increase in pain intensity for 5 of them. After discussion with the research team and the medical team, we modified the task and instructed participants to continue to climb the floors until they reported a 10% increase or until they reached the 6th floor, the last floor of the hospital. This additional limit was set in order to avoid to continue indefinitely the task with patients who were already in pain at the baseline and which was deemed as a significant effort for FM patients by the medical team. The participants therefore stopped physical activity when they reached an increase of at least 10% in their pain on the Pain Visual Analog Scale-intensity or when they had reached the sixth floor of the hospital.

#### Rumination versus distraction induction

The rumination induction, adapted from Nolen-Hoeksema and Morrow^49^, in French version^50^, was used to induce rumination vs. distraction. Participants were presented with a series of 15 sentences displayed on the screen, each for 40 s. The instructions differed depending on the experimental condition. In the rumination condition, participants were instructed to focus on the causes, consequences and signification of each of the sentences (e.g., “Analyze the causes, the consequences and the signification of the tension in your muscles”, “Analyze the causes, the consequences and the signification of the way you react”, “Analyze the causes, the consequences and the signification of how quick or slow your thinking is right now”). In the distraction condition, participants were asked to imagine a situation or an object (e.g., “Imagine the shape of a large black umbrella”, “Imagine the layout of a typical classroom”). The full task lasted for 10 min in each experimental condition.

#### Positive and negative affective scale—state version^51,52^

This 20-item scale in the French version distinguishes between three components: positive affect, anxiety and dysphoria. (e.g., “Right now, I feel interested”). Participants respond on a five-point Likert scale from 1 (not) to 5 (extremely), to a list of 20 adjectives describing affective states. Higher score on each dimension reflected higher positive affect, higher anxiety or higher dysphoria respectively. The total score on positive affect ranged from 10 to 50 and on dysphoria and anxiety from 5 to 25. Internal consistency for the PANAS dimensions (was satisfactory at all three measurement times (ranging from: *α* = 0.79 to *α* = 0.92).

#### Pain visual analogue scales

Visual Analogue Scales (VAS) were commonly used in research and clinical practice to assess pain in patients. In the present study VAS for the intensity of pain (VAS-intensity) and a VAS for the emotional discomfort linked to pain (VAS-discomfort) scales were used^37^. Intensity and discomfort linked to pain were reported by participants on a 100 mm (coded a posteriori from 0 to 100) scale where the highest score indicates that the pain is judged to be the most intense or causing the highest possible discomfort.

#### Momentary ruminative self-focus inventory (Mor, Marchetti & Koster, unpublished manuscript, 2013)

In this 6-item questionnaire assessing the use of state rumination in a given context, participants responded on a 7-point scale from “strongly disagree” to “strongly agree” To assess their current level of rumination (e.g., “Right now, I am thinking about the possible meaning of the way I feel”). The total score ranges from 6 to 42. This scale was adapted and used in a French population^38^. The internal consistency was good at both measurement times (respectively: *α* = 0.85, *α* = 0.88).

## Statistical analysis plan

First, we checked whether the pain activation procedure had a similar effect in both groups by performing a two-way mixed-design ANOVA 2 (experimental group: distraction, rumination) × 2 (measure time: 1—baseline, 2—after physical activity) on both Pain VASs.

In order to test the rumination vs. distraction induction on pain we planned to perform a two-way mixed-design ANOVA 2(experimental group: distraction, rumination) × 2(measure time: 2—after physical activity, 3—after experimental induction). In order to check this effect on affect, we computed a two-way mixed-design ANOVA 2(experimental group: distraction, rumination) × 2(measure time: 1—baseline, 3—after experimental induction). Additionally, a two-way mixed-design ANOVA 2 (experimental group: distraction, rumination) × 2 (measure time: 1—baseline, 3—after experimental induction) was computed on the MRSI score in order to check the efficiency of experimental manipulation in inducing rumination. The significance threshold was set at *p* < 0.05. All the post-hoc comparisons were performed with Bonferroni correction for multiple testing. The descriptive statistics for dependent variables are presented in Table 2.

**Table 2:** Descriptive statistic – mean and standard deviation for pain, affect, and state rumination measured at time 1, 2 and 3 for each group.

Variable	Rumination induction group			Distraction induction group		
	Mean (SD)			Mean (SD)		
	Time 1	Time 2	Time 3	Time 1	Time 2	Time 3
Pain VAS- intensity	47.27(26.76)	61.23(24.09)	50.77 (28.56)	51.24 (22.27)	61.75(22.04)	39.60 (20.25)
Pain VAS—discomfort	44.23(27.94)	60.36(27.92)	51.23 (29.89)	60.72(24.79)	65.00(25.03)	43.16 (26.19)
Affect -positive (PANAS)	25.68(6.27)	–	26.41 (7.01)	28.24(6.27)	–	30.24(6.38)
Affect- anxiety (PANAS)	11.63(5.67)	–	11.09 (5.32)	10.76(5.02)	–	9.20 (5.18)
Affect- dysphoria (PANAS)	10.09 (5.76)	–	9.95 (5.09)	7.76 (4.13)	–	8.04(5.26)
State rumination (MRSI)	24.50 (9.71)	–	24.41(10.25)	24.60(8.93)	–	23.48(9.96)

Pain Vas—Pain Analogue Visual Scale; PANAS—Positive and Negative Affect Schedule, MRSI—Momentary Rumination State Inventory.

## Results

### The effect of physical activity–manipulation check of pain activation procedure

The mixed design ANOVA with VAS-intensity scores as the dependent variable revealed a significant main effect of time (1—baseline vs. 2—post physical activity) (*F(1, 44)* = 13.75,* p* < 0.001, *η*^*2*^ = 0.238), but the time by group interaction effect was not significant (*F(1, 44)* = 0.366,* p* = 0.549) suggesting that after physical activity pain intensity increased similarly in both groups. It is important to underline that at this stage of the experiment both groups underwent exactly the same procedure (see Fig. 1, measure time 1 and 2). Similar effects were observed for discomfort linked to pain, with the significant effect of time (*F(1, 44)* = 4.718,* p* = 0.035, *η*^*2*^ = 0.097) and non-significant effect of interaction (*F(1, 44)* = 1.604,* p* = 0.212).

### The effect of rumination versus distraction on pain intensity and discomfort linked to pain

**Figure 2:**
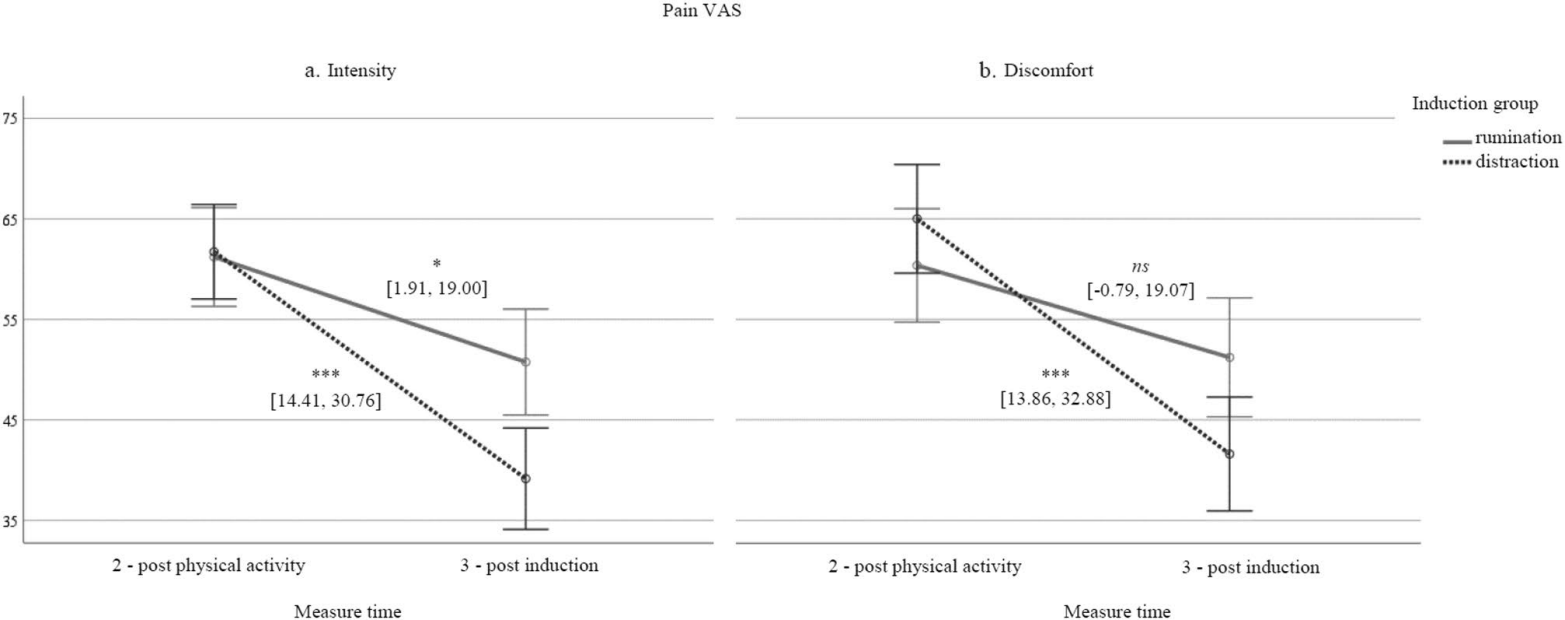
The effect of rumination versus distraction induction on pain intensity and discomfort. *Note*: **p < .05, ***p < .001, ns – not significant; 95% confidence intervals are presented in square brackets.

The mixed design ANOVA with VAS-intensity scores as the dependent variable and induction group (rumination vs. distraction) and measure time (measure time: 2—after physical activity, 3—after experimental induction) revealed a significant main effect of time (*F(1, 44)* = 31.701,* p* < 0.001, *η*^*2*^ = 0.419). The main effect of the group variable was not significant (*F(1, 44)* = 0.747,* p* = 0.392). However, we observed a significant interaction effect between measure time and experimental group (*F(1, 44)* = 4.27, *p* = 0.045,* η*^*2*^ = 0.089). It seems that both induction groups reported a drop in pain intensity, but it was greater among the participants using distraction. The interaction effect with significance level and confidence interval for each slope is presented in Fig. 2a. Similar results were found for discomfort linked to pain, with a main effect of time (*F(1, 44)* = 22.712,* p* < 0.001, *η*^*2*^ = 0.340) and significant interaction effect (*F(1, 44)* = 4.356, *p* = 0.043, *η*^*2*^ = 0.090). It seems that only participants using distraction, not rumination, reported a significant decrease in discomfort linked to pain. The interaction effect with significance level and confidence interval for each slope is presented in Fig. 2b. The main effect of group was not significant (*F(1, 44)* = 0.118,* p* = 0.733).

### Effect of rumination versus distraction on affect

The mixed-design 2(experimental group: distraction, rumination) × 2(measure time: 1—baseline, 3—after experimental induction) ANOVA with general negative affect as dependent variable revealed a non-significant main effect of time (*F(1,45)* = 0.736,* p* = 0.396, *η*^*2*^ = 0.016). Contrary to our prediction, the main effect of group and interaction effects were also not significant (*F*s < 1, *p* > 0.05). The ANOVA with negative affect—anxiety also revealed a non-significant effect of time (*F* < 1, *p* > 0.05), the effect of group and interaction effect were neither significant (*F*s < 1, *p* > 0.05). Similar results were found for negative affect—upset, with non-significant main effect of time, group or interaction effect (*F*s < 1, *p* > 0.05). The ANOVA with the positive affect as outcome revealed no significant main or interaction effects (*F*s < 1, *p* > 0.05).

### The effect of induction on state of rumination

In order to test the experimental manipulation, we performed a mixed-designed ANOVA with MRSI score as outcome. Contrary to our expectation, the interaction effect of time and experimental group was not significant *F*s < 1, *p* > 0.05) which indicates that the change in state rumination between pre and post induction measure did not differ in rumination and distraction group.

## Discussion

According to the partial model of rumination in pain^18^, rumination is causally involved in the maintenance of pain and associated negative affect. One way to break the circle of self-sustaining pain at the cognitive level would be to reduce rumination by engaging in distraction activities that take ones’ attention away from pain^18^. Ruminative thinking might also be one of the processes maintaining avoidance behavior in FM patients^53^. Although the link between rumination and FM is already described in the literature (for a review see:^20^), very little attention has been paid to this process in empirical research in the context of chronic pain, and most of that research has been based on correlational (e.g.,^54^), longitudinal (e.g.^55–57^,) or qualitative studies (e.g.,^19^). Thus, the aim of this study was to experimentally test how rumination induction versus distraction induction impact the evolution of pain and affect in FM patients.

The results of this study provide empirical support for the partial model of rumination^18^. We observed a differential effect of rumination and distraction induction on the change in pain patterns, for both pain intensity and discomfort linked to pain. After performing a physical activity triggering pain, followed by the rumination vs. distraction induction procedure, participants who used distraction report a decrease in pain intensity and discomfort. This effect is significantly lower (for pain intensity) or not observed (for pain discomfort) in the rumination induction group. In other words, rumination interferes with recovery after physical activity in terms of subjective pain, as compared to patients using distraction. These results not only support the partial model of rumination in chronic pain, but are also in line with the literature describing the effects of distraction in pain^32^. However, one interesting result of our study is the lack of effect of rumination vs. induction procedure on patients’ affect (both positive and negative). The results of our induction procedure on pain and affect taken together are particularly appealing when compared to the results of an experimental study by Brookes et al.^58^. They tested the effect of an experimental procedure of rumination versus distraction induction similar to the one used in the present study, on the attentional patterns of undergraduates students who received a threat-inducing information about the cold pressor task^59^. The result suggests that in the trials with shorter exposition, rumination induces a bias toward stimuli linked to pain, while in the trials with longer exposition, it induces experiential avoidance behaviors. These results, alongside the rumination and FM literature, might explain why, on the one hand, rumination might increase the subjective experience of pain through attentional focus on interoceptive stimuli, while on the other hand it does not necessarily have to increase short term negative affect due to its experiential avoidance function. The attentional scope model of rumination^60^ suggest that individuals engaging in ruminative process will struggle to disengage attention from the rumination content. Additionally, a group of studies have already shown that FM is also linked to attentional biases toward pain stimuli^61–63^ and that trait rumination might modify the treatment of pain stimuli also in non-clinical population^21^. Moreover, the FM literature provides some evidence that pain itself might be linked to avoidance tendencies^31,64^. In the present study, rumination at the same time may operate at an overly general, abstract level of processing and might disconnect the patient from their direct emotional experience, even though it is still focused on negative content–pain significance and its consequences^28,29^. This overgeneralization might result in short-term emotional relief, but it also deregulates long-term emotion regulation. In sum, rumination might enclose FM patients in a vicious circle where their attention is sustainably focused on pain stimuli and experience, but at an overly general level, preventing the use of adaptive emotion regulation strategies.

Beyond the aforementioned results, this study was also an opportunity to test the feasibility of an ecological pain activation procedure. This procedure has never been used in the fibromyalgia field. Activating pain to understand its evolution and mechanisms is not a new idea in research. Classically, such activation is conducted using standardized laboratory procedures, for example an activation of C-fibers by thermodes, of A-Beta fibers by pressure^65^, by repeating localized nociceptive stimulation^66^ or by the classical cold pressor task^59^. While pain activation methods may be essential to understand the neurological mechanisms involved in the processing of nociception, such laboratory procedures nevertheless do not reflect chronic pain patients’ everyday life experience, in particular, because they lack an appropriate ecological context. In this study, in order to enable patients to process the pain experience at the cognitive level in ecological setting, we used an everyday-life activity that patients do regularly at home, while working, shopping, etc. All participants who agreed to participate in the research were compliant and completed the task, either until their initial pain increased by 10% or until they reached the sixth floor. We did not record any dropouts. This physical activity allowed a significant increase in pain in our two groups. We did not record any adverse events during our procedure. This is therefore an ecological activity, accessible and easy to set up in a clinical situation, which makes it possible to activate pain without using laboratory equipment.

One of the limitations of the present study is that the measure of rumination (MRSI) did not show any significant differences between the distraction and rumination groups, in spite of the differences reported by participants in their subjective pain intensity and discomfort. One possible explanation for this unexpected result is the choice of the Momentary Ruminative Self-focus Inventory. Although this scale was cited in previous studies as a tool for measuring state rumination^67–70^, its validation has never been published. The tool was published in a modified version as the Brief State Rumination Inventory—BSRI^71^, but unfortunately the data collection in our present study had started prior the publication of BSRI. An alternative relevant solution might also be to use recently validated items assessing rumination in an ecological momentary assessment setting^72^.

The absence of a control group accounting for the spontaneous recovery of pain is also a significant limitation of the present study. It constrains the drawing of conclusions on the causal role of rumination in the maintenance of pain, particularly taking into account that distraction is suggested to have a positive effect on pain recovery^32^. It remains therefore unclear whether rumination really impairs pain recovery or that distraction is an effective method of coping with pain. However, such a control group seems difficult to design, especially given that one can hypothesize that, if left without any precise instruction, patients will use their habitual regulation strategy. Thus, designing a passive control group for this kind of experiment is challenging, and should definitely be the subject of reflection for further studies. Moreover, one of the challenges in the study design was to reduce the amount of cognitive activities (e.g., questionnaires to fill in) between physical activity and rumination vs. distraction induction. Thus, we do not have the data on patients’ affect just after physical activity induction and cannot evaluate whether patients’ emotional reactivity to physical activity impacts further use of rumination or distraction. It would be interesting to consider in future studies alternative methods of evaluating affect that would interfere less with rumination induction, for example passively collecting psychophysiological data.

Additionally, it would be interesting, in further studies, to take into account not only the maladaptive, abstract form of rumination, but to also test the impact of an adaptive concrete experiential rumination processing mode on pain^73,74^. In line with the processing mode theory^73^, some studies have experimentally shown that abstract versus concrete rumination might have a differential impact on affect and emotion regulation^50,74^. Testing this hypothesis in the FM context would be particularly appealing in the light of the lack of a significant impact of the induction procedure on affect in the present study. We can hypothesize that this lack might be linked to emotional avoidance, thus, testing also concrete experiential processing mode might provide an interesting insights into the role of avoidance in FM. Testing the adaptive rumination mode or comparing maladaptive rumination to other strategies, e.g. relaxation, might be also crucial in the perspective of the studies suggesting that distraction, in the long term, can become problematic in chronic pain, as it can also be seen as an avoidance/escape strategy^13,30^.

Despite these limitations, the results obtained are relevant from a clinical point of view and suggest that addressing ruminations in FM treatment could have a direct effect on pain level, but also on avoidance behaviors. Thus, rumination-focused treatments^75^ could be potentially adapted for the management of pain and its comorbidities in FM—particularly given that a key element in FM treatment is to gradually restart an appropriate physical activity. Russel et al.^12^ underlined that FM patients may find themselves in great difficulty facing this activity resumption, which may be associated with a feeling of loss and decreased self-efficiency. The feeling of loss results from the perceived discrepancy between their current condition and their past abilities. The perception of this gap is hypothesized to trigger rumination processes according to control theory^28,76^. Additionally, some studies suggest that kinesiophobia is also linked to a higher level of rumination^11^. Therefore, it seems necessary to support the resumption of physical activity in FM by also anticipating the risk of engaging in rumination which could alter the physical activity benefits, but also patients’ self-efficiency. Moreover, underlining the role of cognitive processes when training teachers of adapted physical activity could limit the risks of post-activity rumination and potentially reduce avoidance mechanisms. This path seems particularly promising considering that the first study testing Rumination-Focused Cognitive Behavioral Therapy in chronic pain patients (chronic low back pain) suggests that it might reduce depression, anxiety and pain severity^77^.

In conclusion, participants with FM who engage in a process of rumination following a physical activity seem to recover less from their pain experience compared to those who engage in a distracting activity. These results endorse Edwards’ partial model of ruminations in pain^18^, which highlights the causal role of rumination in the development and maintenance of pain and its comorbidities. If this causality is confirmed in larger studies, psychological and physical FM treatment will have to evolve to better take into account this cognitive process of pain maintenance.

## Data Availability

The datasets generated and analysed during the current study are available from the corresponding author, MK.
